# Assessment of experimental OpenCV tracking algorithms for ultrasound videos

**DOI:** 10.1038/s41598-023-30930-3

**Published:** 2023-04-25

**Authors:** A. A. Levin, D. D. Klimov, A. A. Nechunaev, L. S. Prokhorenko, D. S. Mishchenkov, A. G. Nosova, D. A. Astakhov, Y. V. Poduraev, D. N. Panchenkov

**Affiliations:** 1 Moscow State University of Medicine and Dentistry Named After A.I. Evdokimov, 20/1 Delegatskaya ul., Moscow, Russian Federation 127473; 2 Moscow State University of Technology “STANKIN”, 1 Vadkovsky per., Moscow, Russian Federation 127055

**Keywords:** Surgical oncology, Biomedical engineering

## Abstract

This study aims to compare the tracking algorithms provided by the OpenCV library to use on ultrasound video. Despite the widespread application of this computer vision library, few works describe the attempts to use it to track the movement of liver tumors on ultrasound video. Movements of the neoplasms caused by the patient`s breath interfere with the positioning of the instruments during the process of biopsy and radio-frequency ablation. The main hypothesis of the experiment was that tracking neoplasms and correcting the position of the manipulator in case of using robotic-assisted surgery will allow positioning the instruments more precisely. Another goal of the experiment was to check if it is possible to ensure real-time tracking with at least 25 processed frames per second for standard definition video. OpenCV version 4.5.0 was used with 7 tracking algorithms from the extra modules package. They are: Boosting, CSRT, KCF, MedianFlow, MIL, MOSSE, TLD. More than 5600 frames of standard definition were processed during the experiment. Analysis of the results shows that two algorithms—CSRT and KCF—could solve the problem of tumor tracking. They lead the test with 70% and more of Intersection over Union and more than 85% successful searches. They could also be used in real-time processing with an average processing speed of up to frames per second in CSRT and 100 + frames per second for KCF. Tracking results reach the average deviation between centers of neoplasms to 2 mm and maximum deviation less than 5 mm. This experiment also shows that no frames made CSRT and KCF algorithms fail simultaneously. So, the hypothesis for future work is combining these algorithms to work together, with one of them—CSRT—as support for the KCF tracker on the rarely failed frames.

## Introduction

Neoplasms detected in the human liver are quite diverse. They can be parasitic invasions—echinococcus or alveococcus, non-parasitic cysts—both solitary and multiple, including those caused by polycystic disease, liver abscesses and inflammatory pseudotumors, liver tumors^1^. Tumors can be benign or malignant. Malignant tumors, in turn, can be primary—hepatocellular or cholangiocellular cancer and metastatic—colorectal metastases, metastases of pancreatic cancer, lung cancer, breast cancer, stomach cancer^2^.

A liver adenoma is a benign liver tumor that occurs in 1 case in a million cases. An incidence increase was noted in women receiving oral contraceptives^3^. Measuring less than 5 cm, liver adenomas, for the most part, do not have specific symptoms. Focal nodular hyperplasia refers to hamartomas and is associated with congenital or acquired vascular malformations leading to hepatocyte hyperplasia. It has an asymptomatic course with a size of less than 5 cm. Iso- or hypoechoic formation during Doppler scanning, which makes it possible to find a central supply vessel, diverging in the form of a spoked wheel to the periphery^3^. Diagnosis of simple liver cysts is not so difficult. They usually occur in women 50 + years and are fluid formations that are well detected by ultrasound, CT, or MRI^1^. Biliary cystadenomas are similar in their histological structure to mucinous adenomas of the pancreas and differ from simple liver cysts by the presence of septa, layering, papillary protrusions, and calcification. The most common liver formations are hemangiomas, up to 20% of the total population. Hemangiomas have a specific ultrasound or CT-MRI structure with a characteristic accumulation of contrast^4^.

Abscesses are secondary cystic lesions of the liver. As a rule, they are associated with biliary infections accompanying obstruction of the biliary tract or manipulation of the bile ducts or parasite infestations^5^.

Primary liver cancer ranks 5th in the incidence of malignant neoplasms in the United States with a 5-year survival rate of 20%; colorectal cancer ranks 3rd, while mortality from it is also in 3rd place in the structure of malignant neoplasms^6^. This can be explained by the fact that 20% of initially diagnosed cases are determined already at the 4th stage with liver metastases^7^. In gastric cancer, the diagnosis of the disease at the 4th stage is noted in one third of all cases^8^, while liver metastases in these patients were noted in 41.30%^9^. It is also significant that the frequency of metastasis of various malignant neoplasms depends on age. The most common source of liver metastases is breast cancer for women aged 20–50 and colorectal cancer for men. As patients get older, a more heterogeneous group of cancers with liver metastases emerges, including cancers of the esophagus, stomach, small intestine, melanoma, bladder cancer, in addition to a significant proportion of lung and pancreatic cancers. The 1-year survival rate of all patients with liver metastases is 15.1% compared to 24.0% in patients with non-liver metastases. Regression analysis showed that the presence of liver metastases causes a decrease in survival, especially in patients with cancer of the testicles, prostate, breast, and anus, as well as in patients with melanoma^10^.

In recent years, minimally invasive surgery has become the standard in cancer treatment^11^. It reduces the period of hospitalization and postoperative recovery. Modern minimally invasive surgery also implies the widespread use of technological advances such as robotic manipulators^12^, computer vision systems^13^, and artificial intelligence^14^. Modern research spot, that using robotic systems for carrying out minimally invasive surgical procedures significantly increases their quality and efficiency^15^. In particular, this is due to the fact that modern robotic systems can achieve higher accuracy parameters than allowed by natural human systems.

Minimally invasive ultrasound-guided interventions can be divided into diagnostic, therapeutic-diagnostic, and therapeutic groups. Diagnostic ones include biopsies of liver tumors, taking fluid from the cavities of the cysts to clarify the diagnosis. Treatment and diagnostic include manipulations when the diagnostic stage immediately precedes the treatment. Therapeutic measures include various manipulations aimed at cure process—drainage of abscesses, bile ducts if they are obstructed, methods of local destruction of liver tumors, such as radiofrequency ablation (RFA), cryoablation, microwave ablation (MVA), irreversible electroporation of tumors. These interventions require high precision during the operation phase. Firstly, due to the determination of safe access to the dilated duct, tumor, or cyst and to the fact that therapeutic effects on liver tumors may be accompanied by thermal damage. This requires precision installation of the working parts of the electrodes, avoiding close contact with the vascular and secretory structures of the liver^16^.

Some studies use state-of-the-art technologies for ultrasound image analysis. For example, distinguishing hepatocellular carcinoma with contrast-enhanced ultrasound describes excellent performance sonographic method^17^. There is also a Feature Fusion method for diagnosing atypical hepatocellular carcinoma in contrast- enhanced ultrasound^18^. Another study describes multi-view patterns for diagnosing hepatocellular carcinoma^19^.

Computer-aided diagnosis (CAD) technology based on Deep Learning and toolkits like VTK^20^ and ITK^21^ help vertebra modeling^22^, and these toolkits could be used for ultrasound diagnosis.

Movements and deformations of the abdominal organs caused by breathing and other processes lead to a deviation of the neoplasm's target position from the preliminary plan of the operation based on CT or MRI data. Thus, in the case of using robotic systems in minimally invasive surgery, there is a need for intraoperative navigation, which can provide real-time data of the target's position for automated control of the medical instrument. There is also a study on developing a deep-learning model for respiratory motion estimation in ultrasound sequences with a deviation of less than 1 mm^23^.

One of the simplest and most common methods of intraoperative visualization of the abdominal organs is ultrasound diagnostic. Its main advantage over intraoperative computed tomography and magnetic resonance imaging is greater availability due to a lower price. For example, the cost of an intraoperative MRI device from leading European manufacturers exceeds a million € while ultrasound devices, which by their characteristics allow solving such problems, have ten times less cost. Also, in comparison with computed tomography, it does not adversely affect the patient and operating personnel. This can be very important in cases of long-term operations. However, it should be noted that ultrasound images are often inferior in quality of computed tomography due to the deterioration or lack of clear visualization of tissues caused by ultrasound artifacts which can negatively impact the interpretation of the results. The detection and dynamics of neoplasms using ultrasound are not an easy task for medical practitioners. Applying modern computer vision technologies could reduce the labor costs of medical personnel and possibly increase the accuracy of determining the center of neoplasms needed for real-time navigation of robotic manipulators for such operations as biopsy and radiofrequency ablation.

## Technical background

The main goal of this work was to test publicly available algorithms for tracking objects within an ultrasounds video. They are under heavy development and are presented in experimental branch of OpenCV modules. Both quality and performance were investigated.

The OpenCV library is the standard for developing computer vision applications. This project was launched in 1999 by the research division of Intel Corporation. For more than 20 years of development, the library has been replenished with many modules that find their application in such areas as face and gesture recognition, robotics, objects detection and segmentation in an image, augmented reality, and many others. This library is open-source software licensed under the Apache 2.0 License. Another reason for choosing OpenCV was the support for a tracking module with a wide variety of different algorithms, high-quality documentation, and a large user community.

Since the OpenCV library is widely used in many areas, the community created certain photo and video data sets to analyze the quality and speed of included algorithms^24^. In particular, there are data sets for testing tracking algorithms^25^. Usually, these sets include various objects of the real world (people, animals, objects) captured on video with variable quality (presence of noise, insufficient illumination etc.). However, most researchers and programmers widely use such sets that do not contain specific data like ultrasound video images.

Despite there being many Deep Learning based software for tracking objects like MDNET, ROLO, SiamFC etc., this study aims to compare trackers supported with OpenCV Tracker Interface for two reasons. Firstly, any Deep Learning-based tracker needs a large, manually marked dataset to train the network. Secondly, all those trackers require additional steps to integrate with the OpenCV application. Testing different Deep Learning algorithms for tracking ultrasound video is another challenging task.

## Design and methods

OpenCV library version 4.5.0^26^ was used for the experiment. It was compiled from source code with additional modules support^27^**,** including the tracking module. The following algorithms of this module were tested:

- Boosting—based on AdaBoost algorithm with HAAR cascade detector. The main idea of online boosting is the introduction of the so-called selectors. They are randomly initialized, and each of them holds a separate feature pool of weak classifiers. When a new training sample arrives, the weak classifiers of each selector are updated. The best weak classifier (having the lowest error) is selected, where the error of the weak classifier is estimated from samples seen so far^28^.
- MIL—a tracker that is similar to Boosting but also uses a small area around the tracker’s current location^29^.
- KCF—a modified tracker—since the positive samples used in the MIL tracker have large overlapping regions. Processing these regions allows to simultaneously increase the speed and accuracy of tracking^30^.
- CSRT—Correlation Filter with Channel and Spatial Reliability. The spatial reliability map adapts the filter support to the object suitable for tracking, which overcomes both the problems of circular shift enabling an arbitrary search range and the limitations related to the rectangular shape assumption. The spatial reliability map is estimated using the output of a graph labeling problem solved efficiently in each frame^31^.
- MOSSE is a tracker based upon the Minimum Output Sum of Squared Error filter, robust to variations in lighting, scale, and deformations. It can pause and resume when the object is left off and appears again^32^.
- MedianFlow—the main idea is tracking points inside a bounding box by Lucas-Kanade tracker, which generates a sparse motion flow between Image N and Image N + 1. The quality of the point predictions is estimated, and each point is assigned an error. The worst 50% of the predictions are filtered out, while the remaining predictions are used to estimate the displacement of the whole bounding box^33^.
- TLD—Tracking-Learning-Detection—a framework designed for long-term tracking of an unknown object in a video stream. Tracker estimates the object’s motion between consecutive frames under the assumption that the frame-to-frame motion is limited, and the object is visible. The tracker is likely to fail and never recover if the object moves out of the camera view. The detector treats every frame as independent and performs full scanning of the image to localize all appearances that have been observed and learned in the past. Learning monitors the performance of both tracker and detector, estimates detector errors, and generates training examples to avoid these errors in the future^34^.

Despite attempts to apply supervised machine learning methods to solve this problem^35^, such experiments require many video files with reference labeling. This is one of the further works for applying artificial intelligence algorithms for ultrasound video processing. This work considers only the built-in algorithms of the OpenCV library.

In addition, the problem of real-time tracking is often solved by reducing the image fed to the tracking functions with the subsequent restoration of the original size^36^, which is primarily caused by low performance, especially when processing HD images. This technology is not used for the experiment—the frames of the original size frames are examined since a hypothesis is put forward for testing the possibility of analyzing SD images by modern computing systems in real-time. The main idea is to evaluate the performance of algorithms on images of actual size, excluding the reasons that may lead to a loss of tracking accuracy.

For the experiment, 17 anonymized video files were recorded from various ultrasound systems during radiofrequency ablation procedures—9 men and 8 women aged 45–66 years. The following ultrasound systems were used:

1. GE Healthcare LOGIQ e
2. Philips iu22
3. Philips Affiniti 50
4. BK Medical flexFocus 400

The duration of the files ranged from 20 to 30 s with a frame rate of 10–15 frames per second. So, in each file there were from 260 to 430 SD-resolution frames. All video files were clipped so every frame contains the tumor, then every frame was saved to lossless Poratable Network Graphic format to eliminate compression artefacts. At last, all the frames were cropped, not resized, to the same resolution of 700 * 600 pixels so only ultrasound picture remains with no additional information provided by video file, like date, time, patient name, transducer parameters etc. Video parameters are shown in the Table 1.

**Table 1:** Video files specifications.

File name	US system	Duration (s)	FPS	Frames	Original resolution (px)	Transducer	cm/px scale
1.avi	GE Healthcare LOGIQ e	22	15	334	684 * 528	GE 4C-RS	0.023
2.avi		20		304			
3.avi		22		336			
4.avi		24		364			
5.avi		25		375			
6.avi	Philips iu22	28	10	281	800 * 600	Philips C5-1	0.032
7.avi		27		274			
8.avi		28		281			
9.avi		29		294		Philips C5-2	0.036
10.avi		26		264			
11.avi	Philips Affiniti 50	20	15	305	1024 * 768	Philips C5-1	0.032
12.avi		22		338			
13.avi	BK Medical flexFocus 400	21	15	318	1020 * 818	BK 8815	0.118
14.avi		24		362			
15.avi		26		395			
16.avi		25		376			
17.avi		28		423			

The experiment was planned as follows:

1. Preparation of anonymous video recordings.
2. Ground truth labeling.
3. Testing tracker algorithms for video recordings.
4. Analysis of the results.

A small software toolkit was created for the convenience of data processing with several utilities such as:

1. Video frames counter (experiments showed that the OpenCV library incorrectly determines the number of frames of an ASF-stream in a WMV container).
2. A module for saving a sequence of video frames to PNG image format (a format that allows storing video frames without further loss of quality).
3. A module for manual labeling reference areas and saving their coordinates.

The video preparation consisted of taking time-lapse images with the subsequent labeling of the zones to search for. Video frames with manually labeled search areas were taken as the reference coordinates of the neoplasms. Qualified oncologist surgeons have labeled them. Subsequently, these images were analyzed, and the area's coordinates were saved to a log file for the convenient analysis of the tracker’s experiment results.

Since the program for measuring the speed and quality of tracking processed frames works sequentially according to the “frame reading—tracking—data output” scheme. The number of frames per second in the internal representation of the video file did not correlate with the measured indicators since the new frame was processed only after the processing of the previous one had been completed.

Also, since the files were encoded using various codecs (Lagarith, Windows Media Video, etc.), only the time spent by the tracking procedure was considered.

The testing methodology included both quantitative (tracking time) and qualitative criteria of tracking algorithms:

1. Intersection over Union—the main qualitative characteristic—is the ratio of the intersection of the found zone and the ground truth zone to their union^37^. In the case of the ideal operation of the algorithm, these areas will coincide. It is possible to estimate how much the search accuracy varies in percentage in other cases. The resulting value is in the segment [0; 1], which is from 0 to 100%, and the higher the value, the better it shows how much of the ground truth zone is covered by the tracking algorithm.
2. False Positive Percentage (FPP)—the ratio of the found area located outside the ground truth to the whole found area. Allows to find out the percentage of false-positive information.
3. RMSE of Centroids (CD)—the distance in pixels between the center of the found zone and the center of the reference zone. A low value is better as it would precisely position the robotic arm.

**Figure 1:**
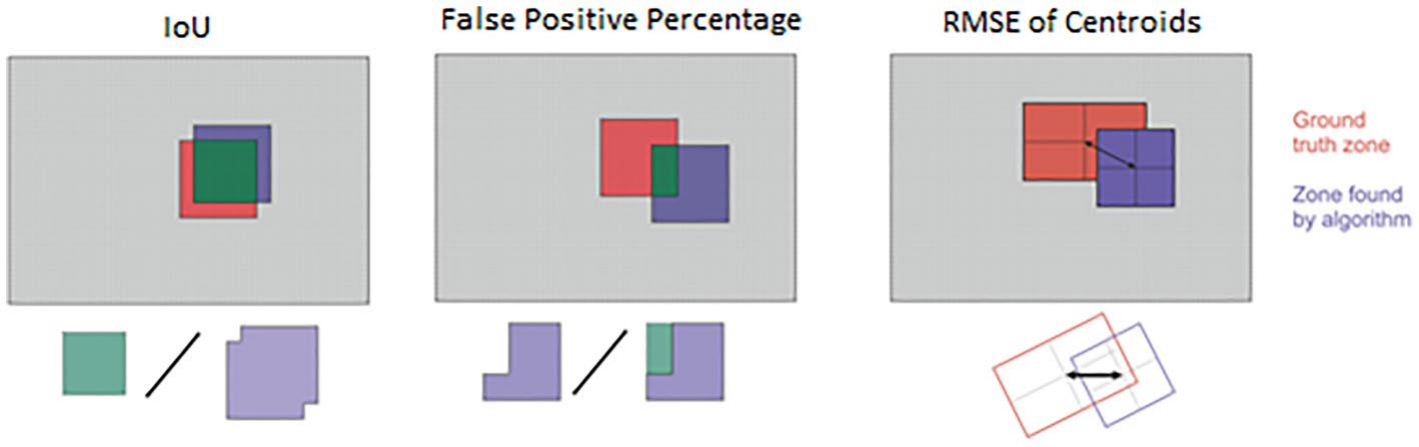
Criteria visual representation.

Figure 1 shows the visual definition of each criterion. In addition, if the best algorithm fails on specific frames, the possibility of re-tracking this frame using another tracking algorithm was checked out^38^.

Every tracker was initialized with the region containing the tumor and all subsequent frames also contained this tumor. Initializing region size varied from 74 * 58 to 198 * 169 pixels. It should allow comparing tracking for frames with different tumor sizes while the whole frame size is the same.

Measuring in pixels in the case of ultrasound videos is more useful to check the quality of trackers as the algorithms know nothing about the tracked object. It is also useful because of different scales in videos, and different resolutions mm per pixel. But in the case of the application for real medical purposes, it is also important to convert pixels to millimeters using the scale of every video.

All software was written in C++ in the Microsoft Visual Studio 2019 development environment.

## Experiment

The experiment was carried out with the following hardware: CPU Intel Core i5-1035G1, RAM 20 GB. Operating system—Ubuntu GNU/Linux 20.04 LTS 64-bits.

The results of the testing program are presented in the Table 2. A total of 5613 video frames were processed.

**Table 2:** Results of trackers testing.

Tracker	Min time (ms)	Avg time (ms)	Max time (ms)	Success/Fails	IoU (%)	FPP (%)	Average deviation of the centroids (px)
Boosting	13	25	64	5613/0	74.36	25.15	12
CSRT	14	22	39	5613/0	68.67	15.41	16
KCF	3	7	15	5512/101	76.93	11.35	11
MIL	1	1	2	2965/2648	67.68	31.57	16
MOSSE	27	31	69	5613/0	48.02	46.65	37
MedianFlow	1	1	1	3516/2097	11.09	47.45	109
TLD	21	38	74	5613/0	43.05	55.13	52

## Results and discussions

A visual inspection of the trackers' work during the testing process made it possible to determine potential favorites and possible outsiders. These ideas were confirmed due to the analysis of the obtained values.

**Figure 2:**
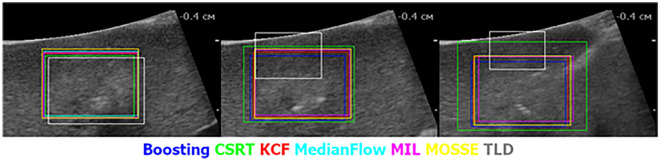
Example of trackers testing.

Figure 2 shows the results of the algorithms processing the file 4.avi. The first frame of the video is on the left pane, frame #100—in the middle, last frame #364—on the right pane. All the algorithms started to work close to each other. However, after 100 frames, the TLD algorithm lost the reference zone, and its error increased by the end of the video series. In addition, the behavior of the CSRT algorithm is indicative—it is the one that managed to process all the frames of the experiment correctly. It tends to increase the search zone area, sometimes capturing excess. Still, at the same time, the centers of the reference and found zones do not diverge so much, which is essential when transferring data to the navigation system of a medical robot. The results of the other algorithms are very similar to each other.

**Figure 3:**
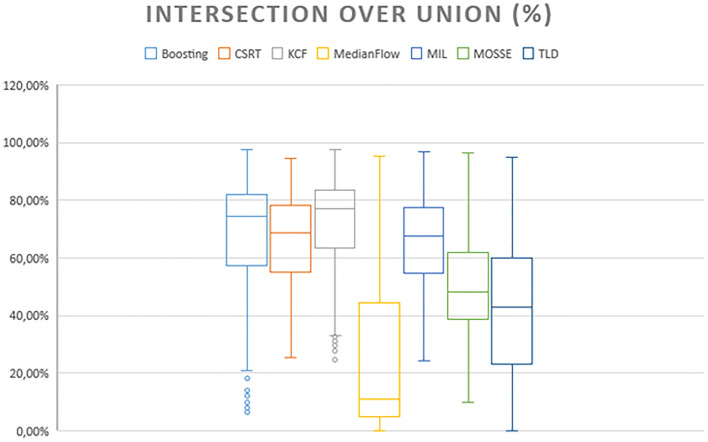
Intersection over Union results.

The Intersection over Union results are shown in Fig. 3. Boosting, CSRT, KCF and MIL trackers are the leaders in this rating, however the results of False Positive Percentage criterion for Boosting and MIL trackers are very poor—up to 50% of founded zone is a false image with no tumor inside for MIL tracker and many outliers for Boosting tracker with poor values. So KCF and CSRT give best results for trackers quality test with FPP rating less than 15%.

*Boosting*—although this algorithm has never reported a negative tracking result, its final results aren’t impressive. In contrast, it has a significant area that is not related to the reference zone. Similar results are shown by the *MOSSE *and *TLD *algorithms—the coverage of the ground truth zone is on an average up to 60%, and the area of the incorrectly defined tumor zone is comparable in size. At the same time, these two algorithms, similarly, to Boosting, always report a successful search result. Besides, they are slower than Boosting on average. It can definitely be concluded that these algorithms are not suitable for solving the problem: both because of the low quality of the results and because of the constant result interpretation as successful.

*MedianFlow* algorithm generally showed similar results. In about 50% of cases, it reported the search failed—which the algorithms described above did not do. However, even in case of success, the area of correct IoU is about 10% of ground truth area with a great area of false-positive data. It is interesting to note the extremely high performance of these two algorithms—the average frame processing time was about 2 ms. This potentially makes it possible to achieve a video analysis speed exceeding several hundred frames per second in case of processing more suitable video.

The problem of some trackers is a constant reporting of «Success Result» while in many cases IoU is equal to zero, so despite the succeeded search no adequate values are founded. For example, it is hard to use Boosting tracker on practice relying only on the tracker messages. Also it is impossible to check the results because of absence of ground truth data for real-time ultrasound images.

*KCF *and *CSRT *lead this test, with an average IoU 70% and more and false-positive results less than 15%.

**Figure 4:**
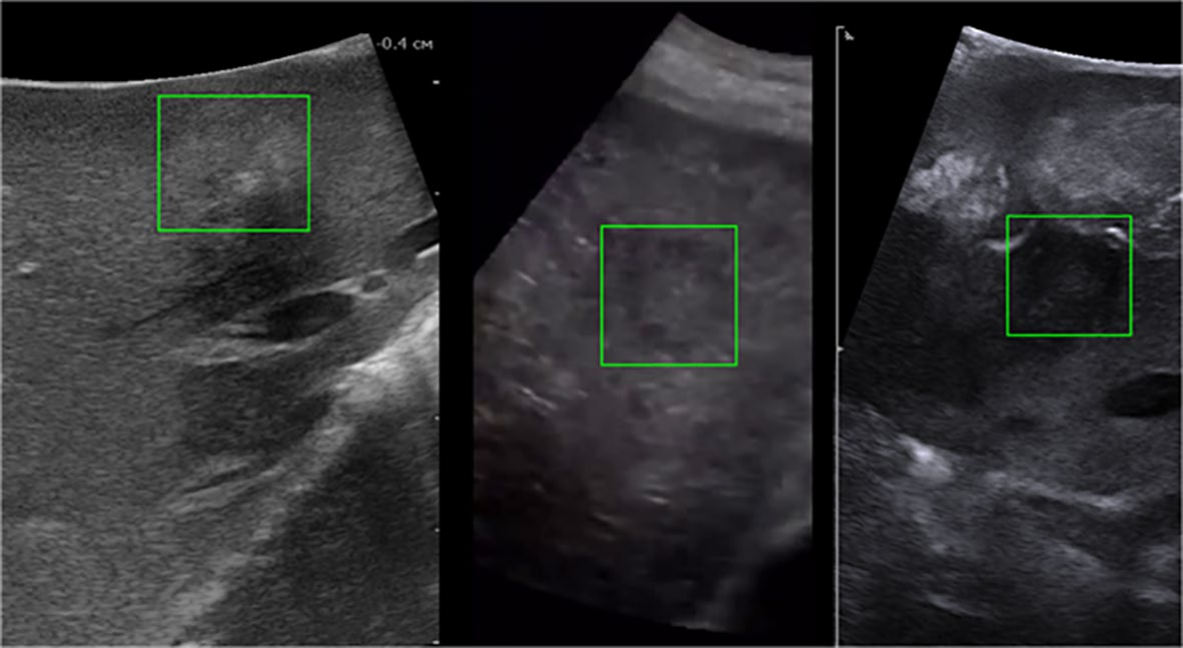
Several frames failed by KCF (Ground truth zone is green).

Some of the frames that failed to process by the KCF algorithm are shown in Fig. 4. CSRT processed all the frames correctly.

The second criterion—Root Mean Square Deviation of Centroids—shows how far the center of the reference zone and the center of the zone found by the algorithms are apart from each other. The closer these centers are located, the more accurately the tracking is made and makes it possible to transmit coordinates for the robotic arm more precisely. The previous two leaders have retained their positions.

False Positive Percentage (Table 2) shows the ratio of the zone outside the reference to the total area of the zone found by the algorithm. It displays the percentage of false information that can lead to a loss of positioning accuracy and should ideally tend to zero. The same leaders remained—KCF and CSRT.

Measuring distances between centers of ground truth and founded zones in pixels makes a sense while developing an application for tracking ultrasound patterns, because all the trackers’ algorithms potentially can track any object in any video. Zooming the ultrasound video will change the size of the pattern on screen, different video will have patterns of different size, but the final result should be presented in measurement system understandable by anybody. Converting pixels to millimeters is a separate process for every video because of different scale. Table 3 shows the root mean square deviation between centers in millimeters.

**Table 3:** Deviation between centers of ground truth and founded zones.

	Average centers deviation (mm)	Maximum deviation (mm)
Boosting	2.59	6.63
CSRT	2.92	7.00
KCF	2.08	5.00
MedianFlow	10.53	24.45
MIL	3.06	7.35
MOSSE	8.49	18.90
TLD	10.98	34.16

**Figure 5:**
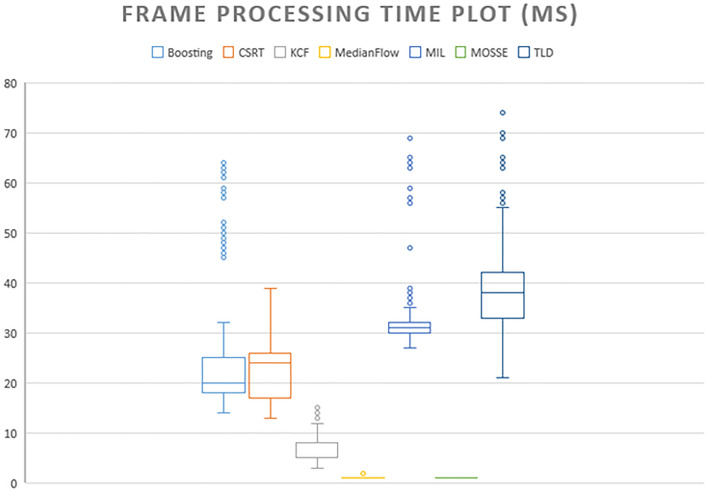
Average frame processing time.

The diagram in Fig. 5 shows that KCF is the only the leader in quality testing; it has excellent speed results. It could make KCF the best choice for tracking lesions on ultrasound video, with the exception of some failed frames.

We have also re-run tests for the assessment leaders—CSRT and KCF algorithms, trying to improve the tracking results. Tuned CSRT initialization parameters are presented below:

- psr_threshold
- padding
- scale_step
- use_gray

KCF parameters that we tuned are presented below:

- detect_thresh
- lambda
- max_patch_size
- sigma

None of them improved the tracking quality, but the quality degradation in many cases during the tuning was perceptible. It means that the developers selected default initialization parameters very carefully. It also should be noted that tracking grayscale or RGB image with CSRT requires the same time to process the frame.

## Conclusions

The CSRT and KCF algorithms are the leaders in this ranking. They always cover the target area at least 70%, and the average result is 75% or more. This result is comparable with Deep Learning methods of tracking ultrasound patterns—SiamFC neural network reaches 83% IoU while tracking carotid artery^39^. False-positive data does not exceed 15% for CSRT and 10% for KCF. The speed of the CSRT algorithm allows it to reach 30 frames per second and KCF—up to 100 frames per second which makes them suitable for real-time processing. The failure rate for the KCF algorithm is less than 2%. With CSRT—all attempts were successful.

The reason for the failures of the other algorithms can be a whole complex of features of the ultrasound image, such as:

- Noisy image,
- The absence of clearly defined contours of objects,
- Gray-scale representation.

The obtained results led to the idea of cooperative use of algorithms: to build a reliable tracking system, it is proposed to use Kernelized Correlation Filters as the main algorithm and in rare cases of its failure to call the Channel Spatial Reliability algorithm, which, despite the lower operation speed, will eliminate dropped frames. The workflow might look like this:

1. Sequential processing of video frames using the KCF tracker.
2. If the KCF tracker fails, the CSRT algorithm is reinitialized with the last successful processed frame and repeats the search on the frame that caused the failure.
3. If step 2 is repeated several (supposedly 3-5 frames) times, the main KCF algorithm is reinitialized with the data received from the backup CSRT tracker.
4. Probably, it makes sense to go to step 2 in case of failure and when the arithmetic means of the distance according to CD criterion exceeds a certain predetermined threshold and/or the value of Intersection over Union criterion exceeds the value of FPP criterion.

The prototype of this application is on heavy development and group of authors hope to publish the result as soon as possible.

Thus, the conclusion of the work suggests that real-time neoplasm tracking is possible using a combination of the two algorithms. This will allow the system to reliably track target areas at frame rates in excess of 50 frames per second. Such development will be future work for this group of authors.

## Data Availability

The datasets analyzed during the current study and results are available via link https://bit.ly/3TrslOt.
